# Identification of novel neuroblastoma biomarkers in urine samples

**DOI:** 10.1038/s41598-021-83619-w

**Published:** 2021-02-18

**Authors:** Kazuki Yokota, Hiroo Uchida, Minoru Sakairi, Mayumi Abe, Yujiro Tanaka, Takahisa Tainaka, Chiyoe Shirota, Wataru Sumida, Kazuo Oshima, Satoshi Makita, Hizuru Amano, Akinari Hinoki

**Affiliations:** 1 Department of Paediatric Surgery, Nagoya University Graduate School of Medicine, 65 Tsurumai, Showa, Nagoya 466-8550 Japan; 2 Hitachi, Ltd., R & D Group, Centre for Exploratory Research, Tokyo, 185-8601 Japan; 3 Department of Rare/Intractable Cancer Analysis Research, Nagoya University Graduate School of Medicine, Nagoya, 466-8550 Japan

**Keywords:** Cancer, Paediatric cancer, Tumour biomarkers, Biomarkers

## Abstract

Urine is a complex liquid containing numerous small molecular metabolites. The ability to non-invasively test for cancer biomarkers in urine is especially beneficial for screening child patients. This study attempted to identify neuroblastoma biomarkers by comprehensively analysing urinary metabolite samples from children. A total of 87 urine samples were collected from 54 participants (15 children with neuroblastoma and 39 without cancer) and used to perform a comprehensive analysis. Urine metabolites were extracted using liquid chromatography/mass spectrometry and analysed by Metabolon, Inc. Biomarker candidates were extracted using the Wilcoxon rank sum test, random forest method (RF), and orthogonal partial least squares discriminant analysis (OPLS-DA). RF identified three important metabolic pathways in 15 samples from children with neuroblastoma. One metabolite was selected from each of the three identified pathways and combined to create a biomarker candidate (3-MTS, CTN, and COR) that represented each of the three pathways; using this candidate, all 15 cases were accurately distinguishable from the control group. Two cases in which known biomarkers were negative tested positive using this new biomarker. Furthermore, the predictive value did not decrease in cases with a low therapeutic effect. This approach could be effectively applied to identify biomarkers for other cancer types.

## Introduction

Childhood cancers are rare, accounting for less than 1% of all cancers, but are the second leading cause of childhood death after road accidents^1^. Therefore, early cancer detection methods are urgently needed. Current approaches include blood tests and imaging studies, such as CT or PET. However, these tests are invasive and involve pain, radiation exposure, and sedation. Recent studies on liquid biopsy have been sequentially conducted using minimally invasive sampling techniques^2^, but generally use blood samples. For children in particular, simple and minimally invasive testing is desirable.

Urine is a complicated and variegated fluid that contains numerous small molecular metabolites^3–5^. There has been significant progress in recent research into the use of urine-derived metabolites for cancer testing, such as polyamines or micro RNA^4,5^. In addition, metabolomics research continues to progress^4,6–12^. Using blood, tissue, bile, and urine samples, metabolomics technology has been used to explore biomarkers for colorectal, breast, bile duct, bladder, and liver cancers^3,4,8–12^. In metabolomics studies, liquid chromatography/mass spectrometry (LC/MS) is often initially used to extract numerous metabolites. Candidate biomarkers are then selected from the many metabolites obtained in the first step. The following method has been successfully used to identify biomarkers for urinary system cancer, breast cancer, and colon cancer^3,4,7,8^. Identified metabolites are first ranked by a machine learning method called the random forest method (RF)^4,9^. Orthogonal partial least squares discriminant analysis (OPLS-DA) is then used to discriminate between groups with or without cancer and evaluate biomarker validity^4,7,9,10,12^. However, all previous studies included only adult patients. Therefore, additional information specific to childhood cancer is required. Neuroblastoma is the most common childhood extracranial malignant solid tumour, comprising between 8 and 10% of all childhood cancers deriving from the adrenal medulla and paravertebral sympathetic ganglia^13^. It is the only childhood cancer for which urinary tumour markers are clinically used^13,14^.

Therefore, this study aimed to establish methods for LC/MS, statistical processing, and machine learning to identify tumour markers for childhood cancers using urinary samples. Since potential new neuroblastoma biomarkers can be verified by comparison with existing markers, neuroblastoma patient samples were used in this study as a representative of childhood cancers. Successful establishment of this method will enable non-invasive and convenient tumour screening and follow-up, thereby increasing the comfort of paediatric patients during examination for cancer.

## Results

### Patients and controls

Fifteen patients with neuroblastoma and 39 controls were included in the study. Patient and control characteristics are shown in Table 1. The control group included preoperative patients, such as patients with inguinal or umbilical hernia. The mean age and ± 2 SD of neuroblastoma patients and control participants were 1.8 ± 3.5 and 2.9 ± 4.1 years of age, respectively.

**Table 1:** Patient and control characteristics.

Variable	Control participants	Neuroblastoma patients	*p *value
Number, n	39	15	
Age, years (mean ± 2SD)	2.9 ± 4.1	1.8 ± 3.5	0.056
Sex, male/female	19/20	8/7	1.000
**Stage (INSS classification)**			
2A, n	(−)	1	
3, n	(−)	1	
4, n	(−)	13	
**Primary tumour localisation**			
Mediastinum, n	(−)	3	
Adrenal gland, n	(−)	10	
Retroperitoneum, n	(−)	2	

*SD* standard deviation, *INSS* International Neuroblastoma Staging System, (−) not applicable.

### LC/MS, Wilcoxon rank sum test, and random forest methods

A total of 998 metabolites were detected by LC/MS. Of the 998 detected metabolites, we extracted 255 metabolites that significantly (P < 0.05) increased or decreased in the neuroblastoma group compared to that in the control group using the Wilcoxon rank sum test. The number of metabolites was decreased to 191 when exogenous substances, such as drugs, were removed from the 255 metabolites. The contribution and importance of the 191 metabolites were ranked using the RF method. Of the top 30 metabolites, a search of the available databases revealed that 11 did not have identified structures; these were therefore excluded. Of the remaining 19 metabolites, 6 significantly decreased in neuroblastoma patients compared to the control group, and 13 were significantly increased. The 19 metabolites are shown in Table 2, along with their associated metabolic pathways. Known tumour markers such as homovanillate (HVA) and vanillylmandelate (VMA) appeared in the top ranked metabolites. Focusing on the metabolic pathways of metabolites that significantly increased in neuroblastoma patients compared to the control group, most highly ranked metabolites (including HVA and VMA) were involved in tyrosine metabolism, methionine metabolism, steroid metabolism, or Leucine metabolism. Based on this information, we believed that it was possible to select biomarker candidates with a higher sensitivity than single metabolites by combining multiple metabolic pathways. We examined various combinations through trial and error. As a result, there were fewer false negatives and higher explained variation (R2) and predictive ability (Q2) when combining substances with different metabolic systems than when combining substances with the same metabolic system. In addition, the combination with a steroid system with a slightly lower ranking had fewer false negatives and a higher R2 and Q2 than the combination with a leucine system with a higher ranking. Therefore, we selected one representative metabolite from each of three different metabolic pathways, tyrosine metabolism and methionine metabolism, which are amino acid metabolism system, and steroid metabolism, which is a lipid metabolism system, as a candidate biomarker. Specifically, 3-methoxytyramine sulphate (3-MTS) was selected as a representative of tyrosine metabolites, cystathionine (CTN) was selected as a representative of methionine metabolites, and cortisol (COR) was selected as a representative of steroid metabolites. The combination of the three metabolites was examined as a tumour marker candidate. Known biomarkers (HVA and VMA) were purposely excluded. However, the combination of HVA and VMA was examined for comparison.

**Table 2:** Nineteen of the highest-ranking metabolites according to their contributions evaluated by the random forest method.

Rank	Metabolites	Fold-change (neuroblastoma/control)	Increase or decrease	p value	Super pathway	Sub pathway
1	homovanillate (HVA)	17	Increase	< 0.01	Amino acid	Tyrosine metabolism
2	3-methoxytyramine sulfate	12	Increase	< 0.01	Amino acid	Tyrosine metabolism
3	vanillylmandelate (VMA)	27	Increase	< 0.01	Amino acid	Tyrosine metabolism
4	vanillactate	31	Increase	< 0.01	Amino acid	Tyrosine metabolism
9	3-methoxy-4-hydroxyphenylglycol	19	Increase	< 0.01	Amino acid	Tyrosine metabolism
10	cystathionine	11	Increase	< 0.01	Amino acid	Methionine metabolism
11	3,4-dihydroxyphenylacetate	19	Increase	< 0.01	Amino acid	Tyrosine metabolism
13	3,4-dihydroxyphenylacetate sulfate	9.3	Increase	< 0.01	Amino acid	Tyrosine metabolism
14	dopamine 3-O-sulfate	7.8	Increase	< 0.01	Amino acid	Tyrosine metabolism
17	dehydroascorbate	0.29	Decrease	0.02	Cofactors	Ascorbate metabolism
18	3-methoxytyrosine	13	Increase	< 0.01	Amino acid	Tyrosine metabolism
19	alpha-hydroxyisovalerate	3.6	Increase	< 0.01	Amino acid	Leucine metabolism
23	N2,N5-diacetylornithine	0.56	Decrease	< 0.01	Amino acid	Arginine metabolism
24	urea	0.77	Decrease	< 0.01	Amino acid	Arginine metabolism
25	cortisol	10	Increase	< 0.01	Lipid	Corticosteroids
26	3-methoxytyramine	10	Increase	< 0.01	Amino acid	Tyrosine metabolism
28	homocitrulline	0.33	Decrease	< 0.01	Amino acid	Arginine metabolism
29	tiglyl carnitine (C5)	0.77	Decrease	0.018	Amino acid	Leucine metabolism
30	xanthurenate	0.49	Decrease	< 0.01	Amino acid	Tryptophan metabolism

### Analysis of the combination of three metabolites using OPLS-DA

**Figure 1:**
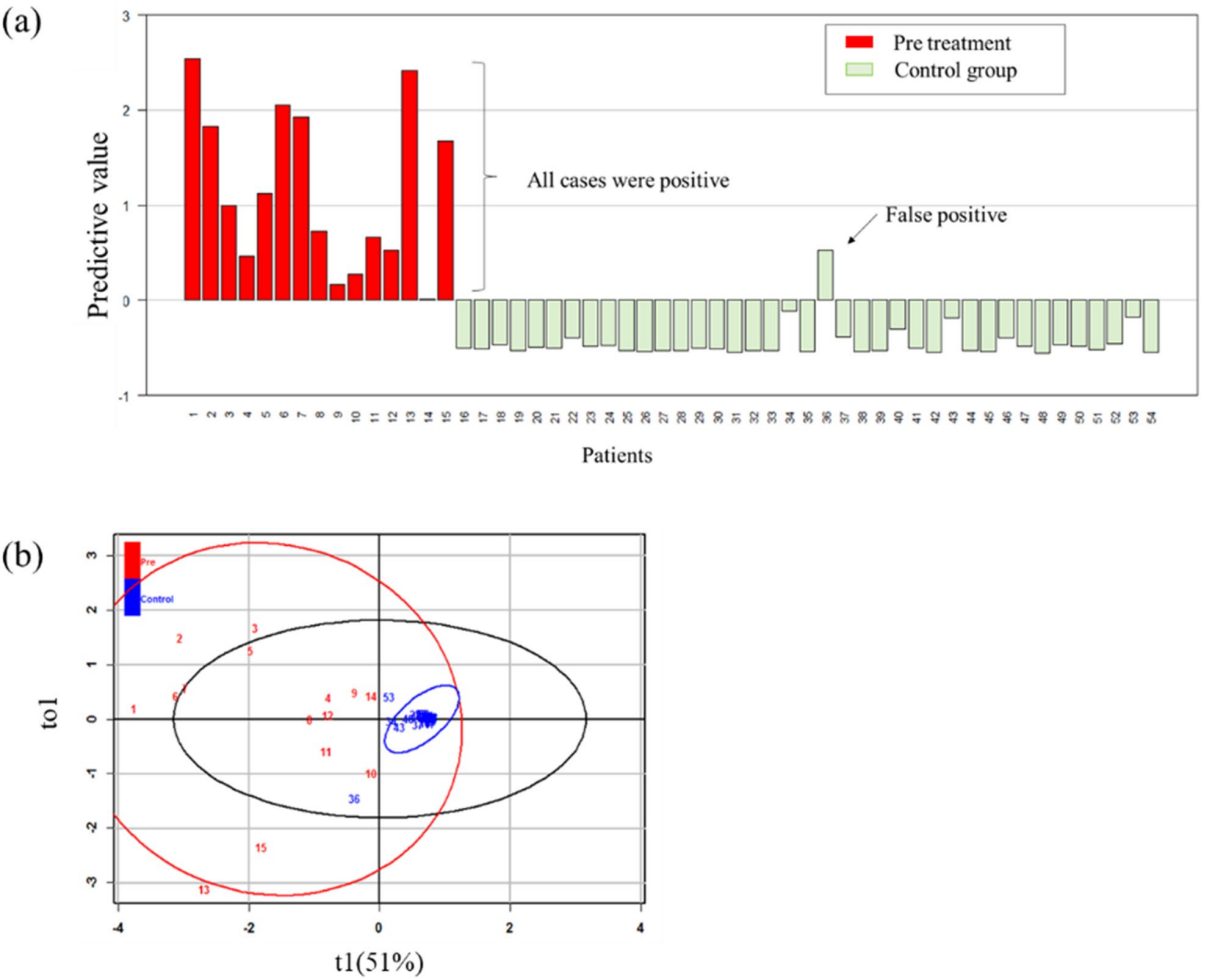
Predictive value analysis of the combinations of three metabolites in pretreatment patients. (**a**) Plot of the predictive values; one bar shows one sample. Positive bars indicate a positive predictive value and negative bars show a negative predictive value. Numbers 1–15 are patients and 16–54 are controls. Patient number 11 was stage 2A and 12 was stage 3. Others were stage 4. (**b**) OPLS-DA results showed that the control group formed a very narrowly scattered pattern and the tumour group formed a widely scattered pattern. The explained variation (R^2^) was 0.726 and predictive ability (Q^2^) was 0.687. These results show that this is an accurate model and that the control group and the tumour group are clearly differentiable.

An OPLS-DA model used the three selected metabolites (3-MTS, CTN, and COR) to discriminate between the neuroblastoma and control groups and determine their respective constants. The calculated constants of 3-MTS, CTN, and COR were 0.52, 0.39, and 0.23, respectively. The predicted value was calculated by adding these constants multiplied by their relative intensities (Fig. 1a). A cut-off value of 0 was designated so that a positive value indicated a high risk of cancer and a negative value indicated a low risk of cancer. The graph showed that all 15 patients with neuroblastoma showed a positive result and one control group patient displayed a false positive result. One patient with a false positive result was seven months old; there were three additional patients aged under one year in the control group who all had negative results but high values. The validity of this result was evaluated by OPLS-DA (Fig. 1b); the control group formed a clustered pattern and the tumour group formed a scattered pattern. R^2^ was 0.726 and Q^2^ was 0.687, which was considered a reasonable result.

### Analysis of the combination of VMA and HVA

**Figure 2:**
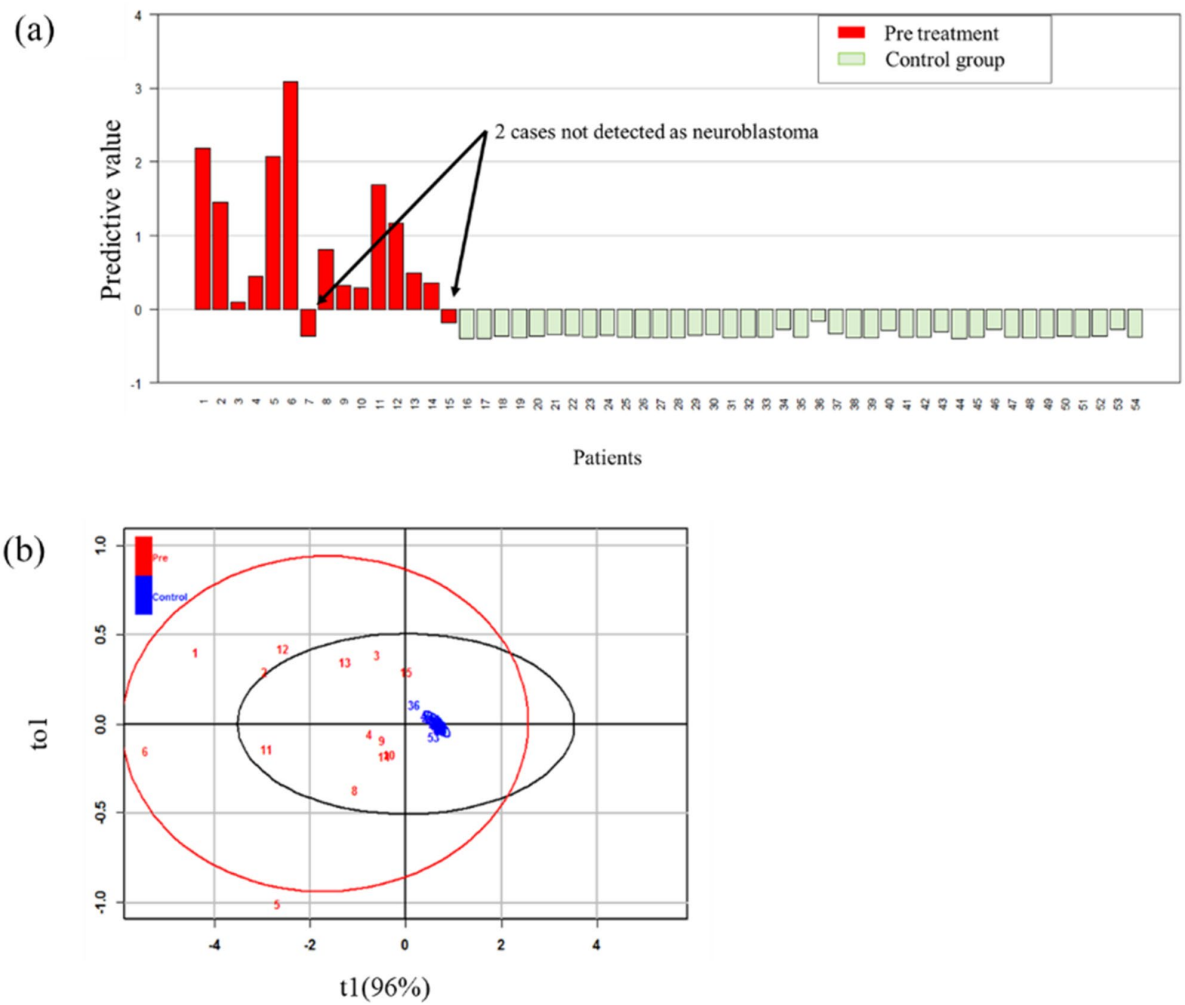
Predictive value analysis of the combination of VMA and HVA in pretreatment patients. (**a**) Plot of predictive values using VMA and HVA. All control group cases were negative. However, in the pretreatment tumour group, two cases were false negatives. Numbers 1–15 are patients and 16–54 are controls. Patient number 11 was stage 2A and 12 was stage 3. Others were stage 4. Two false negative patients were both stage 4. (**b**) OPLS-DA analysis showed that, similar to Fig. 1b, the control group formed a very narrow pattern and the tumour group is out of the graphic. The explained variation (R^2^) was 0.583, and the predictive ability (Q^2^) was 0.512. The control and tumour groups can be clearly discriminated.

We also examined the combination of the known tumour markers, HVA and VMA. The calculated VMA and HVA constants were 0.52 and 0.39, respectively, and the predicted value was calculated for each patient (Fig. 2a). All control group cases were negative. However, in the pretreatment tumour group, two cases were false negatives. In addition, the two cases that were negative for HVA and VMA also had low 3-MTS and CTN levels; only COR was significantly elevated. In the two cases with false negative results, VMA was within the standard clinical value and HVA was slightly higher than the standard value. In OPLS-DA (Fig. 2b), the control group formed a clustered pattern and the tumour group formed a scattered pattern. R^2^ was 0.583 and Q^2^ was 0.512, which was considered a reasonable result.

### Correlation of the predictive value with tumour status after treatment initiation

To evaluate the correlation of the values of the three-metabolite combination with tumour presence or absence, we examined urine samples collected during and after treatment; thirty-three samples were collected from 13 patients. Table 3 shows the correlation between the positive and negative predictive values and the presence or absence of a residual tumour. Tumour presence or absence was judged by ^123^I metaiodobenzylguanidine (MIBG) scintigraphy imaging. There were four false negatives but no false positives. The specificity was 1.000 and the sensitivity was 0.692.

**Table 3:** Correlation between positive and negative predictive values of the combination of novel tumour markers and the presence or absence of a residual tumour.

	Residual tumour ( +), n	Residual tumour (−), n	Sum
Predictive value ( +)	9	0	9
Predictive value (−)	4	20	24
Sum	13	20	33

Similarly, the correlation between HVA and VMA and residual tumour status was analysed. The results are shown in Table 4. There were two false negatives but no false positives. The specificity was 1.000 and the sensitivity was 0.818.

**Table 4:** Correlation between positive and negative predictive values of combination of HVA and VMA and the presence or absence of a residual tumour.

	Residual tumor ( +), n	Residual tumor ( +), n	Sum
Predictive value ( +)	9	0	9
Predictive value (−)	2	20	22
Sum	11	20	31

## Discussion

This study identified a new tumour marker candidate for neuroblastoma that can be identified through non-invasive testing (urine samples). HVA and VMA are known urinary tumour markers for neuroblastoma and their sensitivities are 0.882 and 0.842, respectively^14^. In the current study, although 2 of the 15 pre-treatment patients displayed a false negative for HVA and VMA, all of the novel markers that we identified were positive. Therefore, the markers identified in the current study are not inferior to known markers. Furthermore, R2 and Q2 of HVA and VMA are also relatively low. It may be possible to discover tumour markers that are more sensitive than HVA and VMA, as it is now possible to measure even low concentrations of metabolites. The control group had one false positive result from a 7-month-old patient and four other patients under one year of age. The values in the other three cases were in the negative range but close to the cut-off value of 0, indicating that the dynamics may differ for children under one year of age. In addition, the markers that we identified were useful as indicators of the course of treatment. There were no false positives and the specificity was 1.000. There were four false negatives and the sensitivity was low at 0.692. However, three of the four false negatives came from a single patient. If this patient is considered heterogeneous, the sensitivity reaches 0.900, which is a satisfactory result. Some reports have indicated that combining known markers is effective in identifying additional biomarker candidates^15,16^. This is corroborated by the findings of the current research. The variation in the absolute value of each of the three metabolites was resolved by using a statistical standardisation method^3,17^. The predictive value is the combined value after statistically standardising each of the three metabolites. This method of standardisation is widely used and considered a reasonable statistical procedure^3,7,17,18^.

Metabolomics is a novel and promising tool that has emerged in recent years; it is effective for biomarker identification and discovery^7,10^. The broadest metabolome coverage is achieved by MS-based methods and among MS techniques, LC–MS is the most versatile^7,17^. It is for that reason that we first extracted a wide range of metabolites using LC–MS. Since approximately 1,500 different metabolites were detected by the comprehensive analysis of urinary metabolites, substances that increased or decreased in cancer patients were analysed using the Wilcoxon rank sum test. Even so, 240 metabolites were still considered potential biomarkers. To reduce the number of metabolites, we evaluated the importance of individual metabolites using the RF method, which is an ensemble, supervised machine learning algorithm^4,9,18^. There are many machine learning techniques, but the RF method was adopted because it reduces variance and overfitting, thereby improving accuracy^19^. By excluding substances with an unknown structure, RF analysis further reduced the list of candidates to 20 metabolites. By focusing on the metabolic pathways and selecting one representative substance from each of three different metabolic pathways, we were able to successfully identify novel biomarkers. Both the novel biomarker candidate and the known marker combinations had sufficiently high R^2^ values, indicating a statistically valid and reasonable result^4,7,9,10,12^. Based on this information, the novel biomarker for neuroblastoma was determined to be a combination of 3-MTS, CTN, and COR.

The following sections discuss the relationship between each of these three metabolites and neuroblastoma. First, we considered 3-MTS. Neuroblastoma arises from neural crest cells and a characteristic of neural crest-derived cells is the synthesis of catecholamines such as noradrenalin, adrenalin, and dopamine^20,21^. VMA is the end product of adrenaline and noradrenaline, and HVA is the end product of dopamine^20,22^. Therefore, these have long reigned as known biomarkers. Catecholamine is a metabolite derived from tyrosine^21^. Based on this information, the involvement of tyrosine metabolism in neuroblastoma can be seen. In this study, we chose 3-MTS as a representative of tyrosine metabolites. Several recent studies have evaluated new urinary tumour markers of childhood neuroblastoma and reported 3-MTS as a good marker^22–24^. One report states that 3-MTS is correlated with neuroblastoma stage and prognosis^25^. In our study, all 15 patients had significantly elevated 3-MTS levels before treatment, and 13 of the 15 patients were at stage 4 (Table 1). No correlation with prognosis was found during or after treatment.

We then considered CTN. CTN is an intermediate in the trans-sulphuration pathway of methionine to cysteine^26,27^. Cystathionase, a metabolising enzyme of CTN, requires pyridoxal phosphate (active vitamin B6) as a coenzyme^20,26^. When dopamine is biosynthesised from dopa, dopa decarboxylase acts and requires pyridoxal phosphate as a coenzyme^28^. As described above, catecholamines are overproduced in neuroblastoma. Therefore, in neuroblastoma, pyridoxal phosphate is utilised for dopa decarboxylase activity. As a result, cystathionase activity is reduced and CTN is excreted in large amounts in urine^21,26–28^. Therefore, it makes sense to select CTN as a candidate biomarker. In fact, although there have been no reports in recent years, CTN has been reported as a useful tumour marker for neuroblastoma in the past^21,26,28^. Despite its usefulness, it has not been considered superior to biomarkers such as HVA and VMA. However, it is an important metabolite for neuroblastoma and cannot be excluded when searching for biomarkers using a combination of metabolites, as was the case in our study.

Finally, COR was considered. COR is a type of glucocorticoid (a corticosteroid). Functional adrenal neoplasms such as neuroblastoma, pheochromocytoma, adrenocortical carcinoma, and adrenal adenoma can secrete COR, aldosterone, sex hormones, or catecholamines^29^. There is also a case report of neuroblastoma with elevated cortisol levels^30^. In our study, eight of ten adrenal neuroblastoma patients had significantly elevated COR. COR level did not appear to increase unless the neuroblastoma appeared in the adrenal gland. Therefore, COR appears to be less important for diagnosis than 3-MTS and CTN. However, the two cases that were negative for HVA and VMA may not have been positive for the combination of 3-MTS and CTN alone, and could be extracted with a significant difference by combining COR with 3-MT and CTN.

Based on the above information, the combination of all three metabolites is required to provide a biomarker that is statistically effective and confirms pathology both during and after treatment. Furthermore, these extracted metabolites are all theoretically associated with neuroblastoma. Therefore, we believe that this method of exploring new biomarkers is effective. However, to prove the efficacy of the extracted biomarkers, this marker must be studied in another patient group and a ROC curve must be generated.

The limitations of this study are as follows: First, the number of cases is not large. Significant differences have been observed and the number of populations is statistically sufficient. However, validation using a larger cohort is required in the future. Secondly, there were no cases of early stage neuroblastoma or recurrence. It is important that the extracted biomarkers are able detect tumours early. Recurrence will be considered during follow up on these patients.

In conclusion, we devised an LC/MS and subsequent analysis method to identify new biomarkers for neuroblastoma. Validation of this method focused on three extracted metabolites: 3-MTS, CTN, and COR. The combination of these three metabolites makes them a more useful biomarker than the currently known markers.

In the future, we hope to further validate this new marker for neuroblastoma, while applying this method to identify new biomarkers for other childhood cancers. In particular, we would like to facilitate the detection and identification of tumours for which biomarkers have not yet been discovered. The development of urinary examinations that can detect tumours will improve the ease of diagnosis and follow-up for paediatric cancer patients, because this non-invasive method greatly reduces the discomfort inherent in the currently used methods.

## Methods

### Patients and urinary sample collection

This study recruited paediatric neuroblastoma patients who were treated at Nagoya University Hospital between January 2016 and December 2018. The control group included the patients with no known cancer who were admitted to Nagoya University Hospital for inguinal hernia treatment. After written, informed consent was obtained from each of their parents, urine samples were collected from both patients and controls. If the patient was over 5 years old, they provided informed consent.

Urine samples of neuroblastoma patients were collected once before treatment and then according to the treatment course such as after surgery, chemotherapy, and hematopoietic stem cell transplantation, etc. For the controls, urine samples were only collected once before treatment. The following information was collected from the patient’s medical records: Sex, age, stage, tumour location, treatment, treatment effect, and fluctuation of known biomarkers. Univariate analyses were performed using Fisher’s exact test for sex and the Mann–Whitney U test for age.

### Comprehensive analysis of urinary metabolites

An LC/MS system was used for the comprehensive analysis of urinary metabolites. The multiple isolation mode was selected for LC (reverse phase liquid chromatography and hydrophilic interaction chromatography). This is because LC/MS cannot be limited to a single isolation mode to target metabolites with a wide polarity range for isolation when the substances in urinary metabolites have unknown properties (including being either hydrophilic or hydrophobic or having a charge in solution). We used electrospray ionisation (ESI) in positive and negative ionisation modes (+ ESI mode/-ESI mode) for the MS ionisation method, and a high-resolution Orbitrap mass spectrometer. A high-resolution mass spectrometer enables the refinement of the compositional formula from the precise mass of the ions observed in the obtained mass spectra. This makes it extremely effective for detecting metabolites with unknown structures. The LC/MS analysis was outsourced to Metabolon Inc. (Morrisville, NC, USA), which analysed the metabolites using an original analytical platform and an accepted protocol for LC/MS analysis^4^. However, detailed information about this platform, such as LC gradient conditions, has not been disclosed^4^. The value of each metabolite was expressed in osmotic normalisation value, because components in urine vary widely compared to components in blood.

### Statistical analysis

Metabolites that differed in level significantly in the urine of cancer patients compared to those in the urine of healthy individuals were extracted, and the importance of these metabolites was evaluated. A typical example of this analysis would include creating an S-Plot using the OPLS-DA method; there are reports of methods that extract metabolites with a significant difference in the abundance between healthy individuals and cancer patients with high repeatability^31,32^. However, a large number of urinary metabolites with varying structures exist. Therefore, candidate biomarkers differ for each analysis lot, and it is not always easy to extract biomarker candidates.

Consequently, we adopted the following analysis procedure. We constructed this analysis system independently using the statistical program R version 3.3.2.

1. Comprehensive LC/MS analysis results were refined using p values for metabolites that showed a significant difference between cancer patients and healthy individuals based on the Wilcoxon rank sum test. The level of significance was set at 5%. In this study, the results were obtained by a single test of significance, not a multiple comparison, so correction such as Bonferroni correction or a correction based on the false discovery rate was not used. The variation was so large that normalization could not be performed. Therefore, a nonparametric test was conducted without log-transformation.
2. While it was possible to refine important metabolites using the above testing method, quantitative evaluation was difficult. Therefore, we applied the RF method to the refined analysis targets obtained in step 1 to evaluate metabolite importance^18,19,33^.
3. Biomarker candidates were extracted from metabolites identified as important in step 2. At this point, we excluded conjugates that were estimated to be exogenous metabolites by searching the database owned by Metabolon Inc.
4. We then constructed a cancer detection model based on the OPLS-DA method using the extracted biomarker candidates. A predictive equation represented by the first-order equation of intensities of urinary tumour markers enabled the calculation of the predictive values. For example, in a case with three markers, the predictive value would be calculated as follows:
  $$Predictive \,value = \alpha \times Relative\, intensity\, of\, biomarker\, 1 + \beta \times Relative\, intensity \,of \,biomarker \,2 + \gamma \times Relative\, intensity \,of \,biomarker \,3,$$

where α, β, and γ are constants.

OPLS-DA model quality was evaluated by predicting R^2^ and Q^2^. Q^2^ was calculated by seven cross-validations. A 100-permutation test was used to estimate Q^2^ and R^2^ significance^18^. OPLS-DA revealed that this model successfully calculated the predictive value for that combination and identified valid biomarker candidates.

The predicted values thus obtained were plotted on a graph. This allowed us to compare new biomarker candidates and known markers. For the neuroblastoma group, predicted values were graphed in urinary samples during and after treatment; positive and negative values were determined and summarised in a 2 × 2 contingency table with tumour activity. Tumour presence or absence was judged by MIBG scintigraphy imaging.

### Ethical concerns

This study was approved by the Ethics Committee of Nagoya University Hospital (Approval no: 2016-0303). The legal guardians of all participants provided informed consent. All study procedures were performed in accordance with the guidelines of the Declaration of Helsinki.

## Data Availability

The datasets generated and/or analysed during the current study are available from the corresponding author upon reasonable request.
